# Répercussions métaboliques et cardiovasculaires de la substitution glucocorticoïde au cours de la maladie d’Addison

**DOI:** 10.11604/pamj.2018.30.251.12546

**Published:** 2018-08-06

**Authors:** Dhoha Ben Salah, Nadia Charfi, Mouna Elleuch, Faten Hadj Kacem, Nabila Rekik, Mouna Mnif, Fatma Mnif, Mohamed Abid

**Affiliations:** 1 Service d’Endocrinologie et Diabétologie du CHU Hédi Chaker Sfax, Tunisie

**Keywords:** Maladie d´Addison, hydrocortisone, morbidité, syndrome métabolique, risque cardio-vasculaire, Addison’s disease, hydrocortisone, morbidity, metabolic syndrome, cardiovascular risk

## Abstract

Les études récentes menées chez des patients atteints de maladie d'Addison (MA) ont permis de révéler que cette pathologie, même traitée, reste grevée d'une morbi-mortalité non négligeable. L'objectif de notre étude était de déterminer les effets délétères de la substitution glucocorticoïde au long cours principalement sur le plan métabolique et cardiovasculaire. Il s'agit d'une étude rétrospective qui a inclu 28 patients ayant une MA traitée, évoluant depuis plus que 15 ans. L'âge moyen était de 58,53 ans avec une prédominance féminine à 65%. La durée moyenne de suivi était de 17,87 ans. La dose d'hydrocortisone était initialement à 32,5mg/j (20,52 mg/m^2^) et à 27,9mg/j (16,41mg/m^2^) au moment de l'étude. La prévalence du syndrome métabolique (SM) au cours de la MA était de 35,71% après une durée de traitement supérieur à 15 ans. On note au terme du suivi que 28,57% des patients étaient obèses. Vingt-cinq (25)% des patients avaient développé une HTA et un diabète de type 2. La prévalence de la dyslipidémie était passé de 3,57% à 42,85%. Un seul patient avait présenté un infarctus de myocarde à 25 ans de suivi. Les facteurs favorisant la survenue du SM dans notre étude étaient l'ancienneté de la maladie et la perte du poids à la découverte de la maladie. L'ajustement du traitement substitutif au cours de la maladie d'Addison reste un enjeu au vu de la morbi-mortalité liée au surdosage. Un suivi régulier, et une approche thérapeutique personnalisée sont nécessaires pour améliorer le pronostic de ses patients.

## Abstract (en)

Recent studies conducted in patients with Addison’s disease (AD) highlighted that this disease, even after treatment, is a significant cause of morbi-mortality. This study aims to determine the cardiovascular and metabolic deleterious impact of long-course glucocorticoid substitution therapy. We conducted a retrospective study of 28 patients with treated Addison’s disease evolving for more than 15 years. The average age of patients was 58, 53 years, with a female predominance (65%). The average follow-up period was 17, 87 years. Initial dose of hydrocortisone was 32, 5 mg/day (20.52 mg/m^2^) and 27, 9 mg/day (16,41mg/m^2^) at the time of the study. The prevalence of the metabolic syndrome (MS) in patients with AM was 35.71% after a period of treatment longer than 15 years. At the end of the follow-up period, 28.57% of patients were obese; 25% of patients had developed AH (arterial hypertension) and type 2 diabetes. The prevalence of dyslipidemia went from 3.57% to 42.85%. Only one patient had myocardial infarction at 25-year follow-up. Factors favoring the onset of MS in our study were history of disease and weight loss at the moment of diagnosis. Adjustment of substitution therapy is a challenge in patients with Addison’s disease due to morbi-mortality associated with overdose. A regular follow-up and a personalized therapeutic approach are necessary to improve patients’ prognosis.

## Introduction

L'insuffisance surrénalienne est la conséquence d'un déficit plus ou moins complet de la production et de la sécrétion des hormones corticosurrénaliennes: glucocorticoïdes, minéralocorticoïdes et androgènes. Malgré sa relative rareté, si elle est mal prise en charge, elle peut menacer le pronostic vital. Dans les années 70, l'espérance de vie des patients présentant une insuffisance surrénalienne était considérée comme équivalente à celle de la population générale. Cette notion était remise en cause par de récents travaux suggérant une morbi-mortalité accrue chez les patients présentant une insuffisance surrénalienne [1]. Cette morbidité recouvre à la fois une qualité de vie altérée et certaines pathologies, notamment cardio-métaboliques, pouvant être favorisées par une substitution glucocorticoïde imparfaite [1]. Le traitement substitutif actuel de la carence en cortisol au cours de l'insuffisance surrénale est loin d'être idéal. Une des difficultés réside dans la cinétique de sécrétion physiologique du cortisol, le “cycle nycthéméral”, au cours duquel la sécrétion de cortisol est quasiment nulle vers minuit, démarre en deuxième partie de nuit pour réaliser un pic de sécrétion le matin au lever, puis décroît au cours la journée [2]. Ce cycle est impossible à reproduire avec la forme galénique actuelle de l'hydrocortisone avec sa demi-vie courte. Cette substitution induit donc des taux plasmatiques alternativement supra ou infra physiologiques pouvant être à l'origine d'effets iatrogènes délétères. Les objectifs de notre étude étaient donc d'évaluer le retentissement au long cours de la substitution glucocorticoïde sur le plan métabolique et cardiovasculaire.

## Méthodes

**Patients:** il s'agit d'une étude rétrospective descriptive de 28 patients traités pour maladie d'Addison (MA) depuis plus de 15 ans et suivis dans le service d'Endocrinologie-Diabétologie de l'hôpital Hédi Chaker de Sfax depuis 1977. Le diagnostic était retenu chez nos patients soit par une cortisolémie de base ≤ 30 ng/ml (10 patients), une ACTH > 100 pg/ml (un patient), une cortisolémie 60 min après un test au synacthène immédiat inférieur à 200 ng/ml (18 patients) ou un tableau clinique typique d'insuffisance surrénalienne aigue sans explorations hormonales (9 patients). Nous avons inclus uniquement les patients ayant une insuffisance surrénalienne périphérique d'origine auto-immune.

**Méthodes:** Le recueil des données était fait à partir des dossiers de patients sur des fiches de renseignements. Les paramètres étudiés étaient: l'évolution du poids, de l'IMC et du tour de taille, l'apparition d'une obésité et d'une répartition androïde des graisses, l'apparition d'un diabète de type 2, ou d'une intolérance au glucose au cours du suivi (diabète type 1 exclu), la survenue d'une dyslipidémie, l'apparition d'une HTA, la survenue d'un accident cardiovasculaire, ainsi que la présence d'un (SM) à la dernière consultation. Ensuite nous avons cherché les facteurs favorisant la survenue du syndrome métabolique chez nos patients. Ainsi, nous avons subdivisé nos patients en deux groupes: groupe 1 (G1) avec SM: 10 malades, groupe 2 (G2): sans syndrome métabolique 18 malades. Une corrélation statistique était recherchée avec: l'âge actuel et au moment de diagnostic, le sexe, le niveau socioéconomique et intellectuel, les antécédents personnels et familiaux, la durée de la maladie, le mode de découverte, l'amaigrissement à la découverte de la maladie, le poids l'IMC et la dose d'hydrocortisone.

## Résultats

L'âge moyen au moment de l'étude était de 58,53 ans (extrêmes: 26 à 86 ans). La moyenne d'âge au moment du diagnostic de la maladie d'Addison (MA) était de 37,4 ans (extrêmes: 10 à 69 ans). Les patients étaient repartis en 18 femmes (65%) et 10 hommes (35%). Des antécédents familiaux de maladies auto-immunes étaient présents dans 6,25% des cas et de maladies cardio-metaboliques chez 34,37% des patients. Douze patients, soit 42,8%, avaient des antécédents personnels d'hypothyroïdie, 1 patient (3%) avait une hyperthyroïdie, 3 patients (9%) avaient un diabète type 1 et une patiente (3%) avait une insuffisance ovarienne primitive (IOP). L'insuffisance surrénalienne était découverte essentiellement devant des signes cardinaux chez 62,5% des patients, et à l'occasion d'une insuffisance surrénalienne aigue dans 37,5% des cas. Le recul moyen était de 17,87 ans (extrêmes: 15 à 34 ans). Le traitement était basé sur l'hydrocortisone (cortef ^®^10 mg) chez tous les patients. La dose moyenne était de 32,5 mg/j soit 0,61 mg/kg/j, 20,52 mg/m^2^, repartie en 3 prises journalières, pour la plupart des patients (26 malades). La majorité était sous une dose entre 10 et 20 mg/m^2^. La fluorohydrocortisone (Florinef ^®^) était prescrit chez 28,12% des cas.

**Paramètres anthropométriques initiaux:** Un amaigrissement avant la découverte de la maladie était noté chez 78,12%. La perte de poids moyenne était de 10,6 kg. La mesure du TT était pratiquée chez 4 patients uniquement. Une répartition androïde des graisses était présente chez 2 patientes.

**Etude para clinique:** La glycémie à jeun faite chez 24 patients était en moyenne à 4,8 mmol/l (3- 6,5 mmol/l). Un bilan lipidique initial, réalisé chez 9 patients, était normal chez 8 patients, avec une cholestérolémie moyenne à 3,7 mmol/l (extrêmes 2,89 et 4,9mmol/l), et une triglycéridémie moyenne à 1,35mmol/l (extrêmes 0,65 mmol/l et 2,89 mmol/l). Un patient avait une hyperlipidémie type 2 b (cholestérol à 8,9 mmol/l et triglycérides à 2,72 mmol/l). Il avait une hypothyroïdie de découverte concomitante.

### Evolution et retentissements métaboliques

**Anthropométriques:** On avait noté une prise de poids progressive au cours du suivi pour la plupart des patients (92,85%). Elle était plus marquée au cours de la première année avec une moyenne de 8,79 kg contre une prise de 1,3 kg à la dernière consultation, ainsi qu'une augmentation progressive de l'IMC. La mesure du TT était effectuée chez 4 patients uniquement. Elle était de 99 cm en moyenne à 10 ans de suivi, et de 105,5 cm en moyenne à la dernière consultation. Au terme du suivi, une répartition androïde des graisses était présente chez 3 patients parmi 4.

**Trouble de la tolérance glucidique:** Au cours du suivi, 25% des patients ont développé un diabète de type 2 au bout de 17,14 ans en moyenne (extrêmes 5 et 38 ans). A la dernière consultation 42,8% des diabétiques type2 étaient sous insuline.

**Dyslipidémie:** 42,85% avaient développé une hyperlipidémie après un délai moyen de 18,4 ans (extrêmes 7-38 ans). Il s'agit d'une hypercholestérolémie chez 18,75% des patients, d'une hypertriglycéridémie chez 17,8 % des patients, d'une hypoHDLemie chez 14,28% des femmes (inferieur a 0,5g/l) et 18,18% des hommes (inferieur a 0,4 g/l). Le traitement comportait les règles hygiéno-diététiques, des fibrates dans 50% des cas, et une statine dans 30% cas.

**Hyper tension artérièle (HTA):** 25 % des patients avaient développé une HTA après un délai moyen de 17,4 ans (extrêmes: 12-24 ans). Trois patients parmi eux étaient sous fludrocortisone. Ce traitement était arrêté à la découverte de l'HTA chez deux patients.

**Retentissement cardiovasculaire:** un patient avait présenté un infarctus du myocarde traité par un pontage aorto-coronarien après un délai de suivi de 25 ans.

**Syndrome métabolique:** La prévalence du SM était de 35,71% à la dernière consultation. Le Tableau 1résume les caractéristiques cliniques et biologiques des patients à la découverte de la maladie et au moment de l'étude.

**Tableau 1:** Caractéristiques cliniques et biologiques des patients à la découverte de la maladie et au moment de l’étude

	Initialement	A la dernière consultation
Poids Kg	55,9	71,4
IMC	21,67	27,9
Tour de taille cm	90,25	105,5
Obésité %	7,14	28,5
surpoids %	21,42	78,5
HTA %	0	25
Diabète %	0	25
Glycémie à jeun mmol/l	4,8	8,6
Dyslipidémie %	3,57	42,85
Cholestérol total mmol/l	3,7	4,51
Triglycéride mmol/l	1,35	1,52
HDL mmol/l	-	1,22
LDL mmol/l	-	2,39
SM %	3,57	35,71

**IMC:** indice de la masse corporelle, **SM:** syndrome métabolique

### Facteurs favorisant la survenue du syndrome métabolique

Les facteurs favorisants la survenue du syndrome métabolique dans notre étude étaient l'ancienneté de la MA et la perte du poids à la découverte de la maladie (p=0,05). Pour les autres paramètres, les patients du G1 étaient plus jeunes au moment du diagnostic (p=0,7) et plus âgés au moment de l'étude, pas de corrélation avec le sexe. La majorité étaient analphabètes 70% dans G1 contre 55,6% dans G2 (p=0,46). Ils avaient plus d'antécédents familiaux de maladie cardio-métaboliques. Le poids initial moyen des patients du G1 était plus élevé que celui des patients du G2 (59,3±17,6 kg vs 53,3±11,49 kg) (p=0,28), ainsi que pour l'indice de masse corporelle (23,24±5,94 kg/m^2^ pour G1 vs 21,05±2,99 kg/m^2^ pour G2) (p=0,22). Le groupe 1avait tout le long du suivi un poids et un IMC toujours plus élevés que le groupe 2, mais sans différence significative. Concernant la dose la baisse était similaire chez les deux groupes par rapport à la découverte de la maladie et si elle était plus élevée en mg/j chez le G1 (28,84±5,02 mg vs 27,41±4,52 mg pou G2), elle reste pratiquement la même en mg/kg/j et en mg/m^2^. Le Tableau 2 résume les facteurs favorisant la survenue du SM étudiés chez nos patients.

**Tableau 2:** Facteurs favorisant la survenue du SM

Paramètre	G 1 (n=10) IS avec SM	G 2 (n=18) IS sans SM	P
Age (ans)			
Au moment de dg	35,2 ±13,99	37,17± 14,6	0,7
Age actuel	59,7 ±18,38	56,67±16,81	0,66
Sexe ratio H/F	3/7	8/10	0,46
Analphabète (%)	70	55,6	0,46
Niveau SE médiocre(%)	40	50	0,61
ATCDsF maladies cardio- métaboliques(%)	40	33,3	0,72
HOT (%)	60	27,8	0,1
Ancienneté de l’IS (ans)	25,5 ± 7,9	20,72 ± 4,67	0,05
Mode de découverte IS lente (%)	70	55,6	0,46
Poids initial (kg)	59,3± 17,6	53,3 ± 11,49	0,28
IMC initial (kg/m2)	23,24 ±5,94	21,05± 2,99	0,22
Perte de poids (kg)	14,5 ±7,4	8,3 ±5,9	0,03
Dose d’HC initiale (mg) ; mg/kg/j	35 ±5,27 ; 0,63±0,2	31,67 ±6,18 ; 0,62 ±0,16	0,16 ; 0,86
Dose actuelle (mg) ; mg/kg/j	28,8± 5,02 ; 0,45 ±0,02	27,41 4,52 ; 0,41 ±0,03	0,44 ; 0,86

**G:** groupe, **IS:** insuffisance surrénalienne, **dg:** diagnostic, **H:** homme, **F:** femme, niveau **SE:** niveau socioéconomique, ATCDsF: antécédents familiaux, **HOT:** hypothyroïdie, , **HC:** hydrocortisone, **IMC:** indice de la masse corporelle, **SM:** syndrome métabolique

## Discussion

L'évaluation des effets métaboliques de la substitution glucocorticoïde au cours de la MA et l'étude de ses facteurs favorisants n'ont pas été abordés en Tunisie auparavant. En effet, la sécrétion de cortisol par la surrénale suit un rythme nycthéméral [1]. Dans des conditions normales, les glandes surrénales produisent entre 5 et 10 mg/m^2^ de surface corporelle de cortisol par jour [1], ce qui équivaut à une dose orale de substitution de 15 à 25 mg d'hydrocortisone par jour. Cependant les thérapeutiques actuellement disponibles ne permettent pas de reproduire le cycle nycthéméral physiologique du cortisol, induisant des taux plasmatiques alternativement supra ou infra physiologiques. Ces imperfections peuvent être à l'origine d'effets iatrogènes délétères. La thérapeutique la plus courante est l'hydrocortisone, forme synthétique du cortisol. Elle est disponible sous forme de comprimés à 10 mg. Elle a une demi-vie courte, une biodisponibilité proche de 100% et le pic de cortisolémie est atteint environ 30 minutes après l'ingestion. En raison de cette demi-vie courte il est recommandé donc d'administrer la dose en 2 ou 3 prises par jour [1]. Plus récemment et dans le but de mimer au plus près le profil physiologique des taux de cortisol plasmatique, plusieurs laboratoires pharmaceutiques ont mis au point des formes orales d'hydrocortisone à libération modifiée. Deux formes sont en cours de développement: le Chronocort^®^ et le Duocort^®^ (plenadren) [3]. Selon les dernières recommandations de l'*Endocrine Society* 2016 [4], Il est préconisé d'utiliser l'hydrocortisone á la dose de 15 à 25 mg par jour ou le cortisone acétate (20-35 mg) en deux ou trois prises orales. La dose la plus élevée le matin au réveil, la deuxième au déjeuner, et la troisième l'après-midi. Il est recommandé également d'administrer la deuxième dose au plus tard 6h après la première, afin de minimiser la réduction du cortisol en dessous de la plage normale [5]. Concernant le retentissement au long cours de la substitution glucocorticoïde. Sur le plan métabolique, les données de la littérature concordent sur le risque d'obésité en général [6] et en particulier de type androïde au cours de la MA [7]. Ceci serait dû à une substitution non physiologique par les glucocorticoïdes [8]. L'étude de Leelarathna publiée en 2010 [9], a évalué les comorbidités chez 48 addisoniens. Le surpoids est noté dans 65% des cas dont 23% d'obésité. Ce qui concorde avec notre étude. Les hommes ont un IMC plus élevé que les femmes (29,4 contre 25,8 kg/m^2^, P = 0,029).

L'équipe de H. Filipsson [10] a montré d'autre part, que les patients substitués par des doses d'hydrocortisone supérieures à 20 mg/j ont une augmentation significative de l'IMC. Il est reporté que l'atténuation du rythme circadien du cortisol avec en particulier une cortisolémie matinale basse et une cortisolémie nocturne élevée sont associées à une obésité androïde et au syndrome métabolique [1]. La réduction de l'exposition au cortisol l'après-midi, réduit le poids [11]. En 2009, R. Giordano a évalué le profil métabolique au cours de la MA en comparant 38 addisoniens avec 38 contrôles appariés (même âge, sexe et IMC) [12]. L'ancienneté de la maladie est de 14,6 ans en moyenne (extrêmes 1 et 34 ans). Cette étude a révélé une prévalence plus élevée de répartition androïde (TT> à 102 chez les hommes et 88cm chez les femmes) chez les malades (83%), par rapport aux témoins (44%), en particuliers les femmes. Le TT moyen des malades est de 94,2±2,3 cm, contre 85,8±1,9 cm pour le groupe contrôle. Cette différence est statistiquement significative (p≤0,05). Cependant il n'y a pas de corrélation entre le TT et l'ancienneté de la maladie. Ceci est contradictoire avec d'autres études, qui ont trouvé une corrélation directe entre l'adiposité viscérale avec les doses de glucocorticoïdes et l'ancienneté de la maladie [10, 13]. Concernant le métabolisme glucidique, il est noté un risque plus élevé d'intolérance au glucose voire même de diabète au cours de la MA traitée [6]. En effet, les patients traités par hydrocortisone ont des concentrations de cortisol plus élevées que physiologiquement [6], surtout l'après-midi [14]. Cela réduit chez ces patients, la tolérance au glucose, la sensibilité à l'insuline ainsi que sa sécrétion [15]. R. Berg Thorsdottir a mené une étude observationnelle sur 1675 addisoniens en 2006 au suède [7]. La durée moyenne de la maladie est de 6,5 ans. Il a révélé 12% de diabète de type 2 chez les patients atteints de MA. Cette prévalence est similaire à celle retrouvée dans l'étude de Lovas K en 2002 [16]. Elle est trois à quatre fois plus élevée que la prévalence de 2,7 à 4,3% retrouvée dans la population générale suédoise durant l'année de l'étude [7, 17]. R.Giordano dans son étude, a comparé chez deux groupes, malade et contrôle, la glycémie à jeun, l'insulinémie de base ainsi que la réponse à une hyperglycémie provoquée orale (HGPO) [12]. Il a retrouvé une intolérance au glucose chez 8% des addisoniens. Aucun cas de diabète n'est relevé. La glycémie à jeun moyenne et l'insulinémie de base moyenne sont similaires dans les deux groupes.

Il existe, selon R.Giordano, une liaison positive entre le traitement par glucocorticoïdes et les troubles du métabolisme glucidique [12]. En revanche, aucune corrélation entre ces paramètres glycémiques et la durée du traitement par glucocorticoïdes n'est retrouvée. L'IMC est le meilleur prédicteur d'une réponse anormale à la HGPO puisque tous les patients ayant une HGPO pathologique sont obèses. En effet, chez les sujets sains, la tolérance au glucose est relativement altérée l'après-midi et le soir par rapport au matin [18]. De plus, l'effet immédiat d'une petite augmentation de la cortisolémie sérique, chez ces sujets, est d'inhiber la sécrétion d'insuline [18]. Au cours de la MA, les taux élevés quotidiens de cortisol sérique sont associés à une diminution de la sensibilité à l'insuline. Cette diminution apparait 4 à 6 heures après l'administration de l'hydrocortisone et peut même persister pendant 16 heures [19]. Ceci est illustré par l'analyse de l'effet d'une prise orale de 50 mg d'HC. L'effet hyperglycémiant est plus important lorsque la dose est administrée à 17h par rapport à 5h du matin [19]. Par conséquent, le cycle nycthéméral physiologique du cortisol pourrait en partie être responsable de la variation diurne normale de la tolérance au glucose. Pour cela le remplacement par HC doit mimer la sécrétion physiologique du cortisol pour maintenir l'homéostasie du contrôle glycémique. Dans l'ensemble, on suggère, que des doses faibles de glucocorticoïdes, réduisent l'exposition à l'excès du cortisol surtout l'après-midi, et diminuent les effets délétères métaboliques [20]. Il est prouvé, qu'une diminution des doses d'HC améliore l'hémoglobine glyquée (HBA1C) des patients ayant un diabète concomitant [11]. En ce qui concerne le métabolisme lipidique, il est trouvé une corrélation positive entre le taux plasmatique du LDL-cholestérol et le cortisol plasmatique endogène chez les sujets sains [5, 21, 22]. Au cours de la MA, le traitement substitutif par glucocorticoïde réduit l'activité de la lipase hépatique. Ceci réduit le métabolisme du HDL2 en HDL3, entraînant une augmentation de la concentration du HDL-C. Celle-ci est expliquée aussi par l'augmentation de l'activité de l'Apoprotéine A1 [23]. Egalement, les glucocorticoïdes réduisent l'activité de la lipoprotéine lipase qui entraîne une augmentation de la triglycéridémie [23]. De plus, le traitement réduit le nombre des récepteurs du LDL, ce qui explique l'augmentation du LDL et du cholestérol total [23]. Au cours de la MA, il est noté donc un risque plus élevé de dyslipidémie [6], en particulier une hypercholestérolémie et une hypertriglycéridémie [24]. La réduction des doses de glucocorticoïdes entraîne généralement la réduction du taux de cholestérol et des triglycérides [24].

Dans l'étude de Leelarathna, une dyslipidémie à type d'hypercholestérolémie est notée chez 65% des patients [9], alors que Gurnell et al ont rapporté des profils lipidiques normaux chez 100 patients atteints de la MA [25]. R. Giordano a montré une prévalence plus élevée de dyslipidémie chez les addisoniens que chez les témoins [12]. Une hypercholestérolémie (CT> 2,4g/l) et une hypertriglycéridémie (TG> 1,5/g/l) sont notées chez 18% des malades contre 8% chez les témoins. Ce qui est similaire à notre population. Un taux de LDL élevé (LDL> 1,6 g/l/) est constaté chez 10 % des malades contre 3% des témoins. Tous les patients ont un taux de HDL > à 1mmol/l. Aucune corrélation entre le profil lipidique et la durée du traitement n'est retrouvée dans cette étude. Ceci rend peu probable pour cet auteur, l'influence de l'effet de la substitution chronique en glucocorticoïde. Plus récemment, on a étudié le risque cardio vasculaire chez 147 Addisoniens d'Afrique du Sud contre 147 témoins, recevant une dose moyenne d'HC de 23,8 mg/j soit 0,33mg/j [26]. Une prévalence plus élevée d'hypercholestérolémie (65%), d'hypertriglycéridémie, de hs-CRP élevée et de LDL augmenté (75%), est noté. A l'encontre de l'étude de R. Giordano, le HDLC est bas dans cette étude chez 75% des cas. Aucune corrélation n'est retrouvée entre la dose d'HC et les paramètres lipidiques en dehors du HDLC. Ce dernier est positivement corrélé à la dose d'hydrocortisone. Ce qui est concordant avec d'autres études [22, 27-29]. La coexistence de la MA avec d'autres pathologies, comme l'hypothyroïdie et le diabète, pourrait contribuer aux altérations du profil lipidique observées chez les addisoniens [23]. Sur le plan tensionnel, R. Giordano dans son étude, a trouvé un profil correct chez les addisoniens et les témoins [11]. On peut conclure donc que le traitement substitutif au cours de la MA est associé à certaines, mais pas toutes les caractéristiques du syndrome métabolique (SM) [10]. La dose d'hydrocortisone et l'ancienneté de la maladie sont les deux facteurs favorisant son apparition [30]. On n'a pas trouvé dans la revue de la littérature des études qui ont évalué la relation entre le traitement glucocorticoïde au cours de la MA et l'apparition d'un syndrome métabolique. Toutes les études ont évalué les critères du SM séparément (intolérance au glucose, obésité androïde, dyslipidémie et HTA). Il est démontré une corrélation positive entre la dose d'HC et l'IMC [31], le TT [7, 31], l'intolérance au glucose [6,7] et le taux du HDL-Cholestérol. Par contre l'ancienneté de la maladie est positivement corrélée uniquement avec le TT.

Le rôle métabolique délétère d'un surdosage en glucocorticoïdes est également illustré par les résultats issus de la cohorte de patients hypopituitaires substitués par hormone de croissance de l'étude KIMS [10]. Le risque cardio vasculaire au cours de la MA est largement étudié dans la littérature. Il est retrouvé élevé au cours de la MA [6, 7, 23, 32] et estimé au double par rapport à la population générale [26]. Plusieurs mécanismes sont intriqués. En premier, l'hypercorticisme iatrogène due au surdosage en glucocorticoïdes. En effet, plusieurs études ont mis en évidence une relation positive entre la dose de glucocorticoïdes et les facteurs de risque cardio-vasculaires. Une dose d'hydrocortisone inférieure ou égale à 20 mg/j n'est pas associée à une élévation du risque cardiovasculaire [18]. Les patients recevant ces doses, ont le même profil métabolique que les témoins. Par contre, des doses, de glucocorticoïdes au-dessus de 30 mg d'HC sont associées à une augmentation du risque d'événements cardiovasculaires ultérieurs, y compris les accidents vasculaires cérébraux et l'infarctus du myocarde [33]. Ce surdosage favorisait par ailleurs, l'augmentation de survenue de plusieurs facteurs de risque indépendamment (diabète, HTA, obésité androïde) [7]. De plus, la substitution par de fortes doses de minéralocorticoïdes a des effets délétères sur le système vasculaire et le cœur [34]. Enfin, l'hypothyroïdie fréquemment associée non traitée augmente le risque d'athérosclérose [7]. R. Giordano dans son étude, a trouvé un profil tensionnel correct chez les addisoniens et les témoins, ainsi qu'une épaisseur intima-média comparable [12]. Mais le risque cardiovasculaire chez ces malades reste élevé, devant l'association d'une adiposité centrale et une hyperlipidémie. Notre étude a quelques limites, la petite taille de l'échantillon et le caractère rétrospectif. Le caractère rétrospectif de l'étude a fait que nous avons beaucoup de données manquantes pour certains paramètres en particulier le retentissement osseux qu'on n'a pas pu évaluer, les analyses statistiques sont également limitées par le caractère rétrospectif de l'étude, enfin le caractère rétrospectif et l'absence de groupe témoin limitent également l'analyse des facteurs prédictifs des anomalies métaboliques au cours de la maladie d'Addison.

## Conclusion

Les récentes études menées chez des patients atteints d'insuffisance surrénale chronique ont permis de révéler que cette pathologie, même traitée, était grevée d'une morbidité non négligeable voire d'une surmortalité. Ces données illustrent la nécessité d'améliorer les schémas conventionnels de substitution en glucocorticoïdes. Deux équipes développent des formulations d'hydrocortisone à libération prolongée dont la prise est quotidienne ou biquotidienne et qui reproduisent de manière plus physiologique le rythme de sécrétion du cortisol. Les bénéfices espérés de ces formulations sont une diminution de la morbidité.

### Etat des connaissances actuelles sur le sujet

- Il existe une surmortalité et une morbidité accrue chez les patients présentant une insuffisance surrénalienne traitée;
- Cette morbidité recouvre certaines pathologies, notamment cardio-métaboliques pouvant être favorisées par une substitution glucocorticoïde imparfaite.

### Contribution de notre étude à la connaissance

- Notre étude est à notre connaissance la première étude tunisienne qui s'est intéressée à évaluer les effets secondaires de la substitution glucocorticoïde au cours de la maladie d'Addison traitée depuis plus que 15 ans;
- La recherche des facteurs favorisant la survenue du syndrome métabolique au cours de la maladie d'Addison.

## Conflits d’intérêts

Les auteurs ne déclarent aucun conflit d'intérêts.
